# Complete genome sequence of *Escherichia* phage iGC_PHA_EC001

**DOI:** 10.1128/MRA.00842-23

**Published:** 2023-11-29

**Authors:** Tahsin Khan, Arefeen Haider, Sezanur Rahman, Shovan Basak Moon, Imtiaz Mahamud, Shakhinur Islam Mondal, Anowara Begum, Sudhangshu Kumar Biswas, Mohammad Jubair, Mustafizur Rahman

**Affiliations:** 1 Genome Centre, Infectious Diseases Division, icddr,b, Mohakhali, Dhaka, Bangladesh; 2 Virology Laboratory, Infectious Diseases Division, icddr,b, Mohakhali, Dhaka, Bangladesh; 3 Department of Genetic Engineering and Biotechnology, Shahjalal University of Science Technology, Sylhet, Bangladesh; 4 Department of Microbiology, University of Dhaka, Dhaka, Bangladesh; 5 Department of Biotechnology and Genetic Engineering, Islamic University, Kushtia, Bangladesh; Loyola University Chicago, Chicago, Illinois, USA

**Keywords:** *Escherichia* phage, bacteriophages, lytic bacteriophage, EC001, iPHaGE, *Phapecoctavirus*, endolysins, Bangladesh, phage

## Abstract

Antimicrobial resistance (AMR) in bacteria poses a global health emergency due to limited treatment options. Here, we report a lytic bacteriophage belonging to *Stephanstirmvirinae* family against an AMR *Escherichia coli* (ST2089). *Escherichia* phage iGC_PHA_EC001 is of genus *Phapecoctavirus* and 148,445 bp in length, encoding 269 predicted protein-coding sequences and 10 tRNAs. The phage encodes two lytic proteins containing phage_lysozyme (PF00959.22) and cell wall hydrolase_2 (PF07486.15) as catalytic domains, respectively.

## ANNOUNCEMENT

The rapid emergence of antibiotic resistance in bacteria cornered public health to seek the most effective alternatives (1). The increase of extended-spectrum cephalosporin-resistant Enterobacterales and carbapenem-resistant Enterobacterales is associated with a high mortality rate (2). With limited new antibiotics in the pipeline, searching for alternatives is the crucial factor in combating antimicrobial resistance (3). This resulted in the resurgence of bacteriophage or phage therapy as an alternative to antibiotics (4). icddr,b Genome Center has been running the current study “Phage Hunting and Genomics (iPHaGe)” to identify and characterize potential phages for prophylactic and therapeutic applications. The current study reports the complete genome sequence of *Escherichia* phage iGC_PHA_EC001 capable of lysing extended-spectrum cephalosporin- and carbapenem-resistant *Escherichia coli in vitro*.

**Fig 1:**
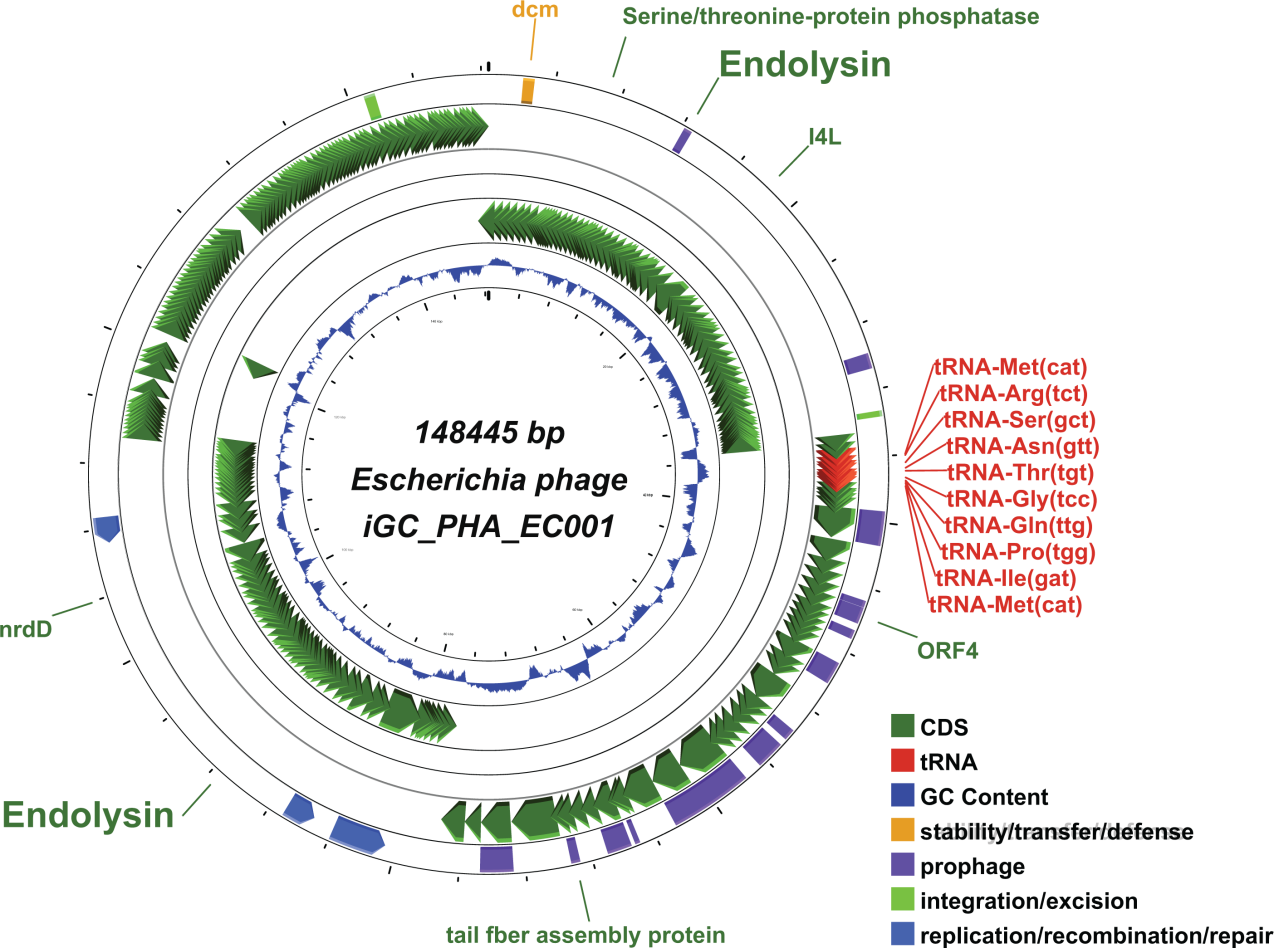
Annotation map of *Escherichia* phage iGC_PHA_EC001 using CGView web server. Positions of endolysins are highlighted in the figure.

A previously characterized clinical isolate *E. coli* ARCH-BD-0001 (Biosample Accession number SAMN35788692) was selected as host for phage hunting in this current study (5). Water samples were obtained in sterile containers from sewage water in Dhaka city. Sample processing and co-culture with *E. coli* ARCH-BD-0001 (ST2089) were done within 3 hours of collection following the methods described in reference (6) (screened with 0.22 *µ*m syringe filter) (6). A single plaque was purified by the double-layer agar method and propagated on the *E. coli* ARCH-BD-0001 isolate. Phage lysate was initially concentrated by PEG8000 precipitation and then DNeasy Blood & Tissue Kit was used with a modified method for DNA extraction (7). The DNA library was prepared using Illumina DNA Prep (Illumina, San Diego, CA, USA) and sequenced in the Illumina MiSeq platform with 500 cycles, yielding a total of 116,621 paired-end (PE) reads. The raw reads were quality controlled using FastQC v0.11.9 (8) and trimmed using trimmomatic v0.39 (9) (SLIDINGWINDOW:4:20 HEADCROP:20 CROP:220 MINLEN:150). *De novo* assembly was performed from 53,927 PE reads with SPAdes v3.15.2 (10) (--careful --only-assembler -k 21,33,55,77,87,99,111,119,127). Contigs shorter than 1,000 bp were assumed to be host contaminants, and the remaining large contig was selected. Assemblies were polished using Pilon v1.24 (11) with “-drags” parameter, and polished assemblies were used to calculate depth coverage using BBmap tool (12). Assembly assessment was checked with Quast v5.2.0 (13), and completeness was confirmed via CheckV v1.0.1 (14). The final length of the assembled genome was 148,445 bp, with a GC content of 39.23% and average coverage of 367.73×. Annotations were performed using Prokka v.1.14.6 (15) in combination with PHROG database (http://s3.climb.ac.uk/ADM_share/all_phrogs.hmm.gz) (16). A total of 269 protein-coding genes were predicted; 79 had putative functions, 190 were hypothetical proteins, and 10 tRNAs (Fig. 1). HMMER v2.41.2 (17) and InterProScan 5.64–96.0 (18) webservers identified phage_lysozyme/glycosyl hydrolase family 24 (PF00959.22), and cell wall hydrolase_2 (PF07486.15) as catalytic domains, respectively. ResFinder and VFDB databases integrated in Abricate v1.0.1 (19) were scanned to determine the presence of antibiotic resistance genes or virulence factors, but none were detected. Bacphlip v0.9.6 (20) analysis predicted the phage to be lytic. NCBI BLASTn (nucleotide collection database) (21) was performed to obtain the closest genomes of iGC_PHA_EC001 and showed similarity with genomes of genus *Phapecoctavirus* under class *Caudoviricetes* (Table 1).

**TABLE 1:** Genome characteristics and comparative analysis of iGC_PHA_EC001 with three closest relatives

Parameter	*Escherichia* phage iGC_PHA_EC001		
Genome Size (bp)	148,445		
Coverage (×)	367.73		
CheckV completeness (%)	100		
CheckV quality	High		
GC content (%)	39.23		
No. CDS	269		
No. of hypothetical proteins	190		
No. of tRNAs	10		
No. of antibiotic resistance genes	0		
No. of bacterial virulence genes	0		
Bacphlip life cycle (%)	Virulent (92.50)		

Closest related phage	*Escherichia* phage ECO340P2	*Escherichia* phage dw-ec	*Escherichia* phage Mt1B1_P17
GenBank Accession no.	OP172792.1	OL739525.1	NC_052662.1
Genus	*Phapecoctavirus*	*Phapecoctavirus*	*Phapecoctavirus*
Query coverage (%)	94	94	93
Percent identity (%)	98.48	98.16	98.4
No. of lytic proteins	02	01	02

## Data Availability

Whole-genome sequencing data are available in the NCBI’s Sequence Read Archive (SRA accession number SRR24776555). The annotated genome assembly is available in NCBI GenBank under accession number OR437326.
